# Are blood groups related to the distribution and severity of COVID-19? A cross-sectional study in a tertiary care hospital in South India

**DOI:** 10.5339/qmj.2021.63

**Published:** 2021-10-21

**Authors:** Elaina Pasangha, Arkadeep Dhali, Christopher D'Souza, Soumya Umesh

**Affiliations:** ^1^St John's Medical College, Bangalore, India E-mail: drsoumya239@gmail.com; ^2^Department of General Medicine, St John's Medical College, Bangalore, India

**Keywords:** COVID-19, coronavirus, ABO blood groups

## Abstract

**Background:** Blood groups are inherited traits that affect the susceptibility/severity of a disease. A clear relationship between coronavirus disease 2019 (COVID-19) and ABO blood groups is yet to be established in the Indian population. This study aimed to demonstrate an association of the distribution and severity of COVID-19 with ABO blood groups. **Methods:** A cross-sectional study was conducted after obtaining ethics approval (IEC 207/20) among hospitalized patients using in-patient records and analyzed on SPSS-25. Chi-square tests were used for the analysis of categorical data and independent sample t-test/Mann–Whitney U tests were used for continuous data. **Results:** The B blood group had the highest prevalence among COVID-19-positive patients. The AB blood group was significantly associated with acute respiratory distress syndrome (ARDS) (*p* = 0.03), sepsis (*p* = 0.02), and septic shock (*p* = 0.02). The O blood group was associated with significantly lower rates of lymphopenia and leucocytosis. However, no significant clinical association was seen in the O blood group. **Conclusion:** This study has demonstrated that blood groups have a similar distribution among patients hospitalized with COVID-19 in the South Indian population. Additionally, it preludes to a possible association between the AB blood group and ARDS, sepsis, and septic shock. Further studies having a larger representation of AB blood groups, especially in patients hospitalized for critical COVID-19, with adjustment for possible covariates, are warranted to provide a reliable estimate of the risk in the South Indian population.

## Introduction

In December 2019 in Wuhan, China, a cluster of cases presented with severe acute respiratory syndrome and was eventually attributed to severe acute respiratory syndrome coronavirus 2 that caused coronavirus disease 2019 (COVID-19)^1^. Since then, the disease has spread across 216 countries and has become a pandemic, with a global prevalence of 156,496,592, resulting in 3,264,143 deaths^2^. A total of 22,295,911 confirmed cases and 242,398 deaths have been reported in India alone, with an average of 378,000 cases per day as of May 2021^3^.

Various epidemiological studies from around the world including India have identified predictors and risk factors associated with clinical characteristics and outcomes of COVID-19^4–7^. Older age, cardiovascular disease, diabetes, chronic respiratory disease, hypertension, and cancer were associated with an increased risk of death^8^. Increased levels of D-dimer, ferritin, lactate dehydrogenase (LDH), and C-reactive protein (CRP) and leukopenia have been identified as laboratory biomarkers of severity^9,10^. Sepsis, ARDS, and septic shock have been classified as critical diseases by the World Health Organization (WHO)^11^.One early study at the Central Hospital of Wuhan reported that the ABO blood groups were associated with susceptibility to COVID-19, with the A blood group having a higher risk for COVID-19^12^.

Blood groups are inherited traits^9^. A link between blood groups and susceptibility to various transmittable respiratory diseases has been described with influenza viruses A and B, rhinoviruses, respiratory syncytial virus, and echoviruses, where the Lewis (Le) blood group antigen phenotypes [Le(a) non-secretor; Le(b) secretor] were compared and secretors were found to be overrepresented in the participants^13^. Blood group antigens may influence disease susceptibility by several mechanisms, such as by functioning as receptors or decoys for infectious organisms and modifying the immune response^14^.

Studies from the USA have demonstrated that the A blood group is associated with a higher risk of COVID-19 infection than non-A blood groups. The O blood group was associated with a lower risk of infection than non-O blood groups^15^. The Rh-negative blood type also appears to have a lesser incidence of outcomes such as intubation, infection, and death^12^.

The mechanisms responsible for this are yet to be elucidated. The most common theory is the presence of neutralizing anti-A antibodies that protect against viral entry by preventing the binding of the virus to the lung epithelium^5^.Studies demonstrating a relationship between COVID-19 and ABO blood groups have given varying results, e.g., Hoilland *et al.* and Zietz *et al.* showed an association of the A/AB blood group with a more severe disease or mortality^15,16^. In two studies conducted in India, Padhi *et al.* illustrated an association between the B blood group and increased mortality from COVID-19 (Spearman r = 0.67, *p* <  0.0001), and Singh *et al.* demonstrated a relationship between the AB blood group and susceptibility to COVID-19 (0.358 vs 0.163 in population, *p* <  0.001)^17,18^. However, there is a paucity of comprehensive data demonstrating a relationship between ABO blood groups and distribution, prognosis, mortality, and morbidity of patients with COVID-19 in India. The Indian Council of Medical Research estimated that India has approximately 70,000 intensive care unit (ICU) beds and even fewer ventilators^19^. In the context of this burdened healthcare system, this study aimed to assess blood groups as a novel component to triaging of both patients and healthcare workers who work in the frontline by investigating the distribution of blood groups among patients with COVID-19 and illustrating a difference in the severity of illness with respect to the ABO blood groups.

## Materials And Methods

After obtaining approval from the Institutional Ethics Committee (IEC 207/2020), a retrospective cross-sectional study was conducted from July to September 2020 among hospitalized patients recruited from the COVID-19 wards, intensive therapy units, and ICUs of St John's Medical College Hospital, Bangalore, a tertiary care teaching hospital. All adult patients who tested positive for COVID-19 by either real-time polymerase chain reaction or a rapid antigen test and whose blood group could be obtained in the in-hospital records were included by convenience sampling. The ABO blood groups were tested by the gel card method, and data were collected. The sample size was estimated based on the findings of Wu *et al.* who reported that 36.9% of patients with a confirmed diagnosis of COVID-19 had blood group type A^20^. Using this estimate of the prevalence with an absolute precision of 5% and 95% confidence interval, the required minimum sample size was 358. However, all 370 patients admitted to the hospital during that time period were included. Adequate precautions were taken during the study to ensure confidentiality of patient records.

Data were collected from hospital records. A waiver of consent was obtained, as it was a record review. Demographic details, presenting symptoms, clinical findings, comorbidities, peak clinical biomarkers such as D-dimer (mg/dl), LDH (IU/l), CRP (mg/dl), and ferritin (mcg/l), and treatment details were captured on a designed structured form.

Data are represented as frequency, mean, or percentage, where indicated. Critical clinical events and peak clinical biomarkers were used to assess severity. Critical clinical events included the need for intensive care, ventilator support (non-invasive ventilation or invasive ventilation), death, and WHO-defined indicators of critical disease, namely, acute respiratory distress syndrome (ARDS), sepsis, and septic shock^11^. These parameters were compared among individual blood groups as well as by grouping those with A antigen and those without, based on the findings of previous studies.^15,16^. Categorical data were analyzed by chi-squared test, and continuous data were analyzed using independent sample t-test or Mann–Whitney U tests, as appropriate. A *p* value of < 0.05 was taken as significant. Statistical analysis was performed using commercially available SPSS version 25.

## Results

### Demographic and clinical characteristics

A total of 370 patients were consecutively recruited from July 2020 to September 2020. A male preponderance was seen, with 186 (50.27%) male patients. The most common symptom was fever (n = 158, 42.7%), followed by shortness of breath (n = 154, 41.62%). The most common comorbidity was hypertension (n = 146, 39.46%), followed by type 2 diabetes mellitus (DM) (n = 141, 38.38%). The majority of the patients were treated with anticoagulants (n = 290, 78.59%) and steroids (n = 250, 67.75%). Approximately 217 (58.65%) patients required oxygen supplementation to maintain saturation >94%. Non-invasive ventilation was required in 91 (24.59%) patients, and 194 (36.22%) patients were intubated. Admission in the ICU was required for 178 (48.11%) patients, and a total of 115 (31.08%) deaths were recorded.

**Figure 1.**
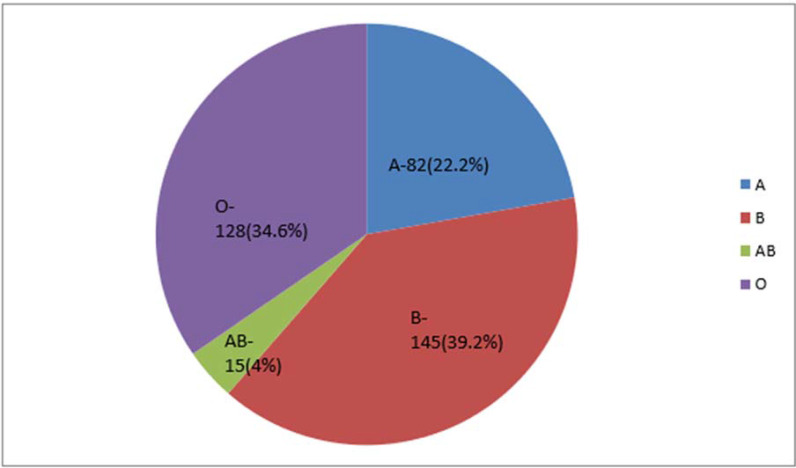
Distribution of the ABO blood groups among the study participants (n = 370)

The B blood group (n = 145, 39.19%) was the most common blood group. The distribution of the ABO blood groups among the patients (N = 370) is illustrated in Figure 1. Rh-positive and negative blood types were present in 347 (93%) and 23 (7%) patients, respectively.

The demographic and clinical parameters were compared between the various ABO blood groups by the chi-squared test. The incidence of type 2 DM (*p* = 0.027) and hypertension (*p* = 0.047**)** were significantly higher in those with A/AB blood groups. Results are illustrated in Table 1.

### Disease severity and ABO blood groups

To assess whether there is a difference in disease severity with respect to the ABO blood groups, we divided the patients into two groups: those with A antigen and those without it. This was performed based on the findings of previous studies where the presence of A antigen was found to be associated with a difference in disease severity. (15,16) However, no significant difference was found in the present study.

Then, we compared each blood group with other groups to elucidate a difference in severity. Regarding disease characteristics, the **AB blood group was found to be significantly associated with worse clinical outcomes including ARDS, sepsis, and septic shock** when compared with other blood groups (A, B, O). Multivariate analysis could not be performed because of the low proportion of patients with the AB blood group. As regards disease characteristics, compared with other blood groups, the O blood group was associated with better clinical biomarkers. The median total leukocyte count (TLC) for the O blood group was 10,500 (IQR, 7340–16450), whereas it was 12,580 (IQR, 9500–19070) for the other blood groups. Lymphopenia was less pronounced in the O blood group (median TLC, 11.4; IQR, 4–19.6) compared with the other blood groups (median TLC, 7; IQR, 1.3–14.1). However, this did not translate to any difference in clinical outcomes.

Similar analysis was performed between A and B blood groups, but no significant difference was found. Results regarding clinical biomarkers are condensed in Table 2, and analysis results regarding clinical outcomes for each blood group are elaborated in Tables 3a, 3b, 3c, and 3d.

Baseline characteristics and outcomes were also compared among Rh-positive and negative blood types. The following outcomes were seen among Rh-positive patients: ARDS (n = 208, 59.9 %; *p* = 0.614), sepsis (n = 142, 62.6%; *p* = 0.629), and septic shock (n = 248, 71.7%; *p* = 1.0). These findings did not show any significant difference.

## Discussion

This study found that the B blood group is the most common blood group in patients hospitalized for COVID-19, which coincides with that found in the general South Indian population, as described by a previous community-based study^21^. This study also showed a significant association of the AB blood group with ARDS, sepsis, and septic shock. However, the A blood group was not associated with the severity of outcomes.

Blood groups are traits that differ in our population because of the founder effects and natural selection^9^. Various studies describing the relationship between COVID-19 and ABO blood groups have demonstrated discordant results. Li *et al.* showed that the A blood group was associated with increased susceptibility to COVID-19, while the O blood group was associated with decreased susceptibility. However, a study conducted in India found that the AB blood group has an increased prevalence of COVID-19^12,18^. In the present study, the B blood group was the most common blood group, which was seen in 39.2% of the study participants. Different theories have been proposed, but the exact mechanisms are yet to be confirmed. One of the theories is that neutralizing anti-A antibodies protect against viral entry into the lung epithelium^13^. Another theory is attributed to the angiotensin-converting enzyme (ACE) receptor, which is involved in pathogen transmission^5^. The GATC haplotype of the four polymorphisms of the ABO gene (i.e., rs8176746, rs8176740, rs495828, and rs12683493), which is prevalent among patients with non-O blood type, is positively associated with the ACE activity^22^.

In the present study, the pattern of distribution of the ABO blood groups was similar to the demography of the South Indian population, with B and O as the most common types and AB as the least common.^20^. This possibly indicates the lack of the relationship between susceptibility to COVID-19 and ABO blood groups. This was similar to that demonstrated in Brazil where no significant association was found between the ABO blood groups and susceptibility to COVID-19^23^.This, however, differed from the results of Zhao *et al.* in their study conducted in Wuhan where patients with the A blood group had a significantly increased chance (OR 1.279) of having an initial positive test compared with those with the O blood group, which had a decreased risk (OR 0.68)^11^.

We compared the salient characteristics of the patients between the blood groups. Type 2 DM and hypertension were significantly higher in those with the A and AB blood groups. This coincides with the results of a study performed in China where the percentage of the A blood group was significantly higher and that of the O blood group was significantly lower in hypertensive patients with coronary artery disease^24^. A clear relationship between type 2 DM and ABO blood groups has not been previously established. No other significant difference in baseline characteristics, including gender, age, or symptomatology, was found among the other blood groups. However, when outcomes indicating severity were compared between the blood groups, the AB blood group was significantly associated with sepsis, ARDS, and septic shock. These three outcomes were taken into consideration because of their association with critical disease as classified by the WHO^2^. This shows that the AB blood group has a worse clinical prognosis. Previous studies in New York by Zietz *et al.* and Canada by Hoiland *et al.* have demonstrated that the AB blood group was associated with increased deaths, increased length of stay in the ICU, greater need for renal replacement, and risk of ventilation^15,16^. However, no studies conducted in India and abroad have compared these severe outcomes (ARDS, sepsis, and septic shock) and the AB blood group. However, a clear association could not be confirmed through multivariate analysis because of the low proportion of patients with the AB blood group in the present study.

Compared with the other blood groups, the O blood group was significantly associated with lower incidence of leucocytosis and lymphopenia. These conditions are among the various clinical biomarkers associated with severe disease^10^. When a trend of laboratory investigations was compared, leukocytosis was postulated to be a consequence of secondary infection and infection-induced cytokine storm^5^. Similarly, lymphopenia was postulated to be not just a consequence of infection but also a probable critical factor driving disease development. B cells, which are responsible for humoral immunity, exhibit the most significant decrease, which led to the failure to limit the release of free virions in the body, resulting in viral expansion^25^. This coincides with other previous studies where the O blood group contained protective biochemical markers against COVID-19^15,16^. However, in the present study, this did not translate to any difference in clinical outcomes for the same.

Mortality was the highest in the B blood group (35.9%), which was similar to the findings of Padhi *et al.* from India^17^. However, this was not significant in the present study.

These conflicting observations on the association of blood group AB with COVID-19 and related ARDS, sepsis, and death could be attributed to the discrepancies in investigated ethnic populations and different strains of COVID-19 with varying pathogenicities. Similar discrepancies have been reported about the relationship between blood groups and *Plasmodium falciparum* in India, which was attributed to a population-specific link^26^. While the relationship between the AB blood group and susceptibility to COVID-19 has been described in a previous study in India, the mechanism of how the AB blood group is associated with the severity of COVID-19 has not been described till date^18^.

Blood groups are a novel, easy, and acceptable method of triaging patients. In the absence of effective targeted therapy and a yet to be widely vaccinated population, triaging is of utmost importance^3^. A study that identifies blood group as a risk factor not only helps in triaging patients but also allows the effective use of healthcare staff as frontline workers, which will ease the stress on the system, especially in India, which is currently facing its biggest COVID crisis. This could serve as a basis for further large-scale studies to establish risk factors for disease.

Furthermore, the present study could provide a platform for research into the relationship between the ABO blood groups and COVID-19 at a molecular level, which could uncover potentially vital information regarding new mutations currently ravaging India. The establishment of a relationship at a molecular or enzyme level could help identify new targets for vaccines and therapy, particularly for severe disease.

As limitation of this study, the sample size was calculated based on the most common blood group, so a larger sample size is required for further analysis, as the AB blood group that is associated with worse outcomes has a low proportion in the South Indian population.

**In conclusion**, the B blood group was the most common blood group among our study participants. The distribution of the ABO blood groups among our study participants was similar to that in the South Indian population, indicating that the ABO blood groups had no role in the susceptibility to COVID-19. Additionally, this preliminary study indicates a possible association of the AB blood group with poor clinical outcomes, such as the development of ARDS, sepsis, and septic shock in patients with COVID-19. However, further studies with a larger representation of the AB blood groups in patients, especially those hospitalized with critical COVID-19, with adjustment for possible covariates, are warranted to provide a reliable estimate of the risk in the South Indian population.

## Figures and Tables

**Table 1:** Comparison of the salient characteristics of the study participants among the ABO blood groups

Characteristic	Total	A	AB	B	O	P value

	N = 370	N = 82	N = 15	N = 145	N = 128	

Sex						0.68

Male	186(50.2)	43(52.4)	7(46.6)	76(52.4)	60(46.8)	

Female	184(49.7)	39(47.5)	8(53.3)	69(47.5)	68(53.1)	

Age (mean in years)*	48.02	48.2( ± 17.6)	49( ± 24.83)	46.1( ± 17.7)	43.3( ± 17.3)	0.09

Comorbidities						

• Hypertension	146(39.4)	39(48.1)	8(53.3)	58(40.0)	41(31.7)	0.03

• Type 2 DM	142(38.3)	39(48.1)	6(40.0)	55(37.9)	42(32.5)	0.05

• CVD	56(15.1)	15(18.5)	1(6.6)	20(13.7)	20(15.5)	0.94

• CVA	34(9.1)	8(9.8)	1(6.6)	17(11.7)	8(6.2)	0.62

• CKD	63(17.1)	19(23.4)	2(13.3)	19(13.1)	23(17.8)	0.14

• Cancer/	51(13.7)	10(12.3)	3(20.0)	19(13.1)	19(14.7)	0.94

Immunosuppression						

• CLD	10(2.7)	0	1(6.6)	6(4.1)	3(2.3)	0.24

WHO clinical classification						

1. Mild	150(40.5)	37(46.1)	4(26.7)	55(37.9)	54(42.2)	0.90

2. Moderate	26(7.1)	4(4.9)	1(6.7)	10(6.9)	11(8.6)	

3. Severe	26(7.1)	6(7.3)	1(6.7)	12(8.3)	7(5.5)	

4. Critical	168(45.4)	35(42.7)	9(60)	68(46.9)	56(43.8)	

Sepsis	138(37.2)	30(37.0)	10(66.6)	49(33.7)	49(37.9)	0.30

Septic shock	104(28.1)	20(24.6)	8(53.3)	42(29.1)	34(26.3)	0.8

ARDS	150(40.5)	31(38.2)	10(66.6)	58(40.0)	51(39.5)	0.61

Pharmacotherapy						

• Antivirals	69(18.6)	10(12.5)	8(53.3)	32(22.3)	19(14.7)	0.97

• Anticoagulants	290(78.3)	68(83.9)	13(86.6)	109(75.6)	100(77.5)	0.11

• Steroids	250(67.5)	55(67.9)	10(66.6)	103(71.5)	82(63.5)	0.99

Oxygen therapy	217(58.6)	44(54.3)	11(73.3)	86(59.3)	76(58.9)	0.75


*mean ±  SD

DM, diabetes mellitus; CVA, cerebrovascular accident; CVD, cardiovascular disease; CKD, chronic kidney disease; CLD, chronic liver disease.

Data are represented as frequency with the percentage enclosed in parenthesis.

**Table 2:** Peak inflammatory markers of the study participants when each blood group was compared with the other blood groups

Biomarker	A versus others: median (IQR)	P value	B versus others: median (IQR)	P value	AB versus others: median (IQR)	P value	O versus others: median (IQR)	P value

CRP (mg/dl)	12.75(4.9–28.7)	0.16	10.8(3.1–25.1)	0.64	4.8(0.2–27.7)	0.21	14.64(1.8–26.4)	0.91

LDH (units/l)	470(282–694.2)	0.32	422(282.7–630.2)	0.45	368(180.5–512.5)	0.47	330(233.6–647.4)	0.17

Ferritin (μg/l)	791(365–1435.3)	0.65	728.8(344.2–1934.2)	0.77	386(239.1–501.1)	0.1	1092(256.7–3362.8)	0.06

D-dimer (ng/ml)	860(386–1425.2)	0.36	1057(557.5–1366.7)	0.70	951(579.5–1359)	0.65	962(471.6–2161.5)	0.85

Leukocyte counts	12250(7860–17015)	0.83	12800(8330–19070)	0.16	13560(9500–17432)	0.47	10900(7340–16450)	0.05

Lymphocyte percentage (%)	7(3.85–13.15)	0.153	7.1(3–18.3)	0.30	5.4(1.6–14.9)	0.16	11.4(4–19.6)	< 0.01


CRP, C-reactive protein; LDH, lactate dehydrogenase; IQR, interquartile range Individual columns give the median value of the biomarker for that respective blood group when it was compared against the others with the interquartile range given in brackets. The P value given beside each blood group is the P value obtained for each t-test when each blood group was compared against the others. Significant results are written in bold

**Table 3d:** Outcomes indicating severity when the O blood group was compared with the other blood groups

Outcomes	O blood group N = 128	Other blood groups N = 242	P value

Oxygen therapy	75(58.6)	142(58.7)	0.98

Non-invasive ventilation	27(21.3)	64(26.7)	0.25

Intubation	41(32)	93(38.4)	0.22

ICU care	57(44.5)	11(45.9)	0.81

ARDS	51(39.8)	99(40.9)	0.84

Sepsis	49(38.3)	89(36.8)	0.77

Septic shock	34(36.6)	70(29)	0.61

Death	39(30.5)	78(31.4)	0.85


Data are represented as frequency with the percentage enclosed in parenthesis.
